# Robustness of Nutrient Signaling Is Maintained by Interconnectivity Between Signal Transduction Pathways

**DOI:** 10.3389/fphys.2018.01964

**Published:** 2019-01-21

**Authors:** Niek Welkenhuysen, Barbara Schnitzer, Linnea Österberg, Marija Cvijovic

**Affiliations:** ^1^Department of Mathematical Sciences, University of Gothenburg, Gothenburg, Sweden; ^2^Department of Mathematical Sciences, Chalmers University of Technology, Gothenburg, Sweden; ^3^Department of Biology and Biological Engineering, Chalmers University of Technology, Gothenburg, Sweden

**Keywords:** nutrient signaling, cAMP-PKA pathway, Snf1 pathway, Snf3/Rgt2 pathway, logic modeling, Boolean logic model, crosstalk

## Abstract

Systems biology approaches provide means to study the interplay between biological processes leading to the mechanistic understanding of the properties of complex biological systems. Here, we developed a vector format rule-based Boolean logic model of the yeast *S. cerevisiae* cAMP-PKA, Snf1, and the Snf3-Rgt2 pathway to better understand the role of crosstalk on network robustness and function. We identified that phosphatases are the common unknown components of the network and that crosstalk from the cAMP-PKA pathway to other pathways plays a critical role in nutrient sensing events. The model was simulated with known crosstalk combinations and subsequent analysis led to the identification of characteristics and impact of pathway interconnections. Our results revealed that the interconnections between the Snf1 and Snf3-Rgt2 pathway led to increased robustness in these signaling pathways. Overall, our approach contributes to the understanding of the function and importance of crosstalk in nutrient signaling.

## 1. Introduction

A biological system can be described as a set of components that interact in such a way that they form a functional unit (Alberghina and Westerhoff, 2005). Systems biology aims to understand the function of the components and how they interact at a systems level. This knowledge about the components provides predictability in the outcome of the system. However, the complexity of many biological processes obstructs the prediction of system outcomes. Mathematical modeling helps to compute the outcome of more complex systems and to identify the properties that emerge from the interaction between the components within the system. This can lead to an improved insight in the mechanistic properties of any biological system.

In signal transduction pathways components can undergo several different changes, such as phosphorylation on multiple sites that are further combined to achieve a subsequent reaction. These are very well-studied through both high-throughput and small scale studies making many components of signaling pathways known (Papin et al., 2005) and providing suitable data for utilizing systems biology approaches by developing a semi-quantitative logic (Boolean) models (Bornholdt, 2008; Wang et al., 2012).

To signal a broad spectrum of nutrients present in the cell environment the yeast *Saccharomyces cerevisiae* has an extensive nutrient sensing network in place. The function of this network is to initiate a comprehensive reprogramming of gene expression to be able to utilize specific nutrients. The yeast carbon and nitrogen sensing systems have been thoroughly studied and their key components have been identified (Gancedo, 2008; Broach, 2012; Conrad et al., 2014; Shashkova et al., 2015; Sanz et al., 2016). However, it is not sufficient just to know the components of a biological system. In order to gain a complete insight into the nutrient sensing system it is necessary to understand the functions of the components and how they interact with each other. In yeast, the carbon source sensing is mainly done by the cAMP-PKA pathway, Snf1 pathway, and the Snf3/Rgt2 pathway. Nitrogen source sensing is performed by the TOR pathway. The knowledge on the functioning of the components and the linearity of these pathways is ambiguous. The ambiguity is due to the substantial amount of crosstalk that has been identified between the components of the different pathways (Broach, 2012; Shashkova et al., 2015; Sanz et al., 2016).

Crosstalk, in biology, is a phenomenon by which an integrated intracellular signal from multiple inputs produces an output that is different from the response triggered by the individual pathways (Vert and Chory, 2011). Two pathways can be interconnected directly by shared component(s), or indirectly when one pathway affects another signaling pathway (Vert and Chory, 2011). The effect of crosstalk on signaling and regulatory pathways has already been studied through mathematical modeling, focusing on the crosstalk from kinases and phosphatases (Rowland et al., 2012, 2015; Rowland and Deeds, 2014). However, the action of kinases and phosphatases embedded in a full network (Endres, 2012) has not been deciphered. In this work we study the direct and indirect crosstalk between nutrient signaling pathways cAMP-PKA, Snf1, and Snf3/Rgt2. Experimental perturbation of these pathways produces noise causing a major challenge in identifying interconnections and therefore theoretical approaches, such as Boolean modeling, are often applied.

Boolean modeling has already been used to reconstruct various signaling pathways (Schlatter et al., 2009; Singh et al., 2012; Anderson et al., 2016). For nutrient sensing pathways a large network reconstruction of the Snf1 pathway has been made based on an exhaustive and manually curated literature review (Lubitz et al., 2015). Further, a logic model describing crosstalk between the Snf1 and Rgt2/Snf3 pathway has been published (Christensen et al., 2009). These however put the emphasis on the technical aspect of modeling of signaling pathways rather than on the predictive possibilities of the Boolean Model.

In this work we aimed to better understand if crosstalk within the yeast nutrient signaling network contributes to the vitality of the nutrient sensing function when the system is perturbed. Specifically, we look at how crosstalk between the Snf1, cAMP-PKA, and Rgt2/Snf3 pathways contribute to the appropriate response to nutritional availability. The model was transformed into a vector format rule-based Boolean model. The created model was completed and validated by a gap filling process based on known input/output relations. We further validated the model by experimental study of protein localization and phosphorylation status. This showed that the model can be used as a tool to predict states of components within the model. Next we included literature curated crosstalk between these pathways. The influence of the crosstalk on the network was evaluated through network perturbation and subsequent analysis of the component states. We found that some crosstalk reactions were vital for the functioning of the network. It was suggested that even in the non-perturbed state they played an important role. Other crosstalk reactions did not have any significant influence on the network output. We further show the modularity of our modeling approach by adding the nitrogen sensing TOR pathway to the model. Overall, we present a Boolean model of a large nutrient signaling network that allows to assess the influence of crosstalk on the network.

## 2. Materials and Methods

### 2.1. Logic Model

**Figure 1:**
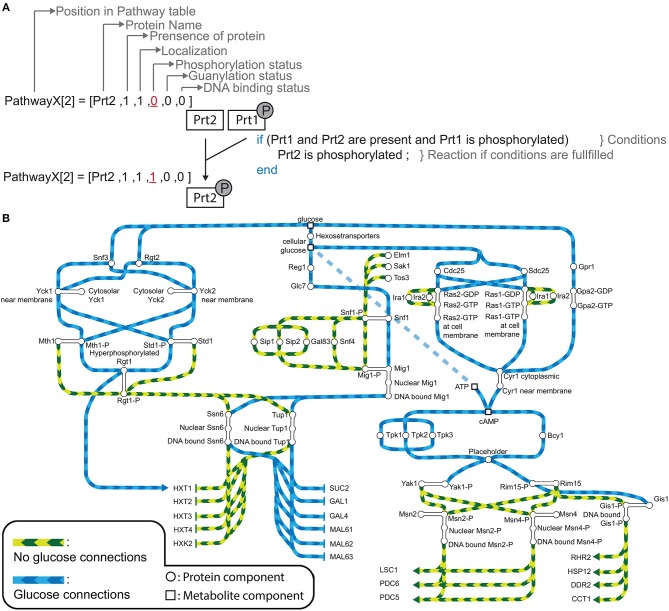
**(A)** An example of a reaction in our model for hypothetical PathwayX, which contains hypothetical components Prt1 and Prt2. Each component is designated a vector, which is a collection of the name and the different states a component can assume. Components Prt1 and Prt2 belong to the table for Pathway X. A reaction in the model only occurs when the conditions described in the if-statement are fulfilled. **(B)** Signal transmission route of “glucose” and “no glucose” conditions through the Rgt2/Snf3, Snf1, and cAMP-PKA pathway and its components. The graph displays the possible states of each component for the system without perturbations (WT-system). Blue lines display the connections between the components when glucose is available. Green lines display the connections between the components when glucose is not available. Round nodes are proteins, square nodes display metabolites, upper-case letters are promoters of genes (e.g., SUC2). An arrow at the end of the lines displays activation of a gene and a vertical stripe gene repression. The connected nodes are the possible states one component can assume.

The model of the nutrient sensing network was based on peer-published literature and each module in the code is denoted with the respective PubMedID of the article (Celenza and Carlson, 1989; Broach, 1991, 2012; Mitts et al., 1991; Kuroda et al., 1993; Haney and Broach, 1994; Hu et al., 1995; Ozcan and Johnston, 1995; Treitel and Carlson, 1995; Martinez-Pastor et al., 1996; Ozcan et al., 1996; Schmitt and McEntee, 1996; Colombo et al., 1998, 2004; Gorner et al., 1998; Lutfiyya et al., 1998; Frolova et al., 1999; Pedruzzi et al., 2000; Schmidt and McCartney, 2000; Jacinto et al., 2001; Düvel et al., 2003; Flick et al., 2003; Kim et al., 2003; Mosley et al., 2003; Cameroni et al., 2004; Moriya and Johnston, 2004; De Wever et al., 2005; Hong et al., 2005; Palomino et al., 2005; Roosen et al., 2005; Swinnen et al., 2006; Peeters et al., 2007; Lee et al., 2008, 2011, 2013; Rubenstein et al., 2008; Georis et al., 2009; Tate et al., 2010; Loewith and Hall, 2011; Orzechowski Westholm et al., 2012; Bontron et al., 2013; Hughes Hallett et al., 2014; Ma et al., 2014; Kayikci and Nielsen, 2015; Shashkova et al., 2017). The model (Figure 1B) was translated to a Boolean logic model and implemented in MATLAB^©^ (The MathWorks, Inc.). In our model there are three types of components: metabolites, proteins and complex components. Each protein is assigned a state vector with six entries defining its name, presence, localization, phosphorylation status, GDP/GTP exchange status, and DNA binding status. A component can: (A) be present or absent, (B) be localized to the membrane, the cytosol or the nucleus, (C) have phosphorylation or guanosine groups, and (D) be bound to DNA. The second type of component, metabolites, are treated in the same manner, however, they only need three properties and therefore their state vector has only length three. Here, phosphorylation, GDP/GTP exchange, and DNA binding are redundant. In some reactions protein complexes are formed. Those are denoted by complex formation components with vector length one and indicate if the complex is active or not.

In the implementation all parameters in the state vector are translated to a bound set of integer values (Tables S3, S4), which are not necessarily purely Boolean but can include more possible outcomes. Each vector uniquely represents one state in the set of all possible states. The components are ordered according to the pathway they belong to (Tables S2, S6). In total, the model comprises 4 metabolites, 63 proteins (including 6 unknown) in 4 pathways, and 19 target genes.

The initial model inputs are the metabolites glucose and nitrogen that can be set to present (1) or absent (0). Starting from that assumption, the information propagates through the pathways by numerous logical operations constructed based on the literature review. Biologically, most modifications are equivalent to activation or inhibition through phosphorylation/ dephosphorylation or GDP/GTP exchange. Figure 1A shows an example for an operation involving two proteins in an arbitrary pathway XXXpw: if protein 2 is present (XXXpw{2,2} == 1) AND protein 1 is present (XXXpw{1,2} == 1) AND phosphorylated (XXXpw{1,4} == 1), protein 2 gets phosphorylated (XXXpw{2,4} = 1). The phosphorylation status of protein 2 therefore increases from 0 to 1.

In the model a typical operation is therefore a change in the state vector of a component that only happens under certain conditions (rules for an reaction to happen). Conditions are usually composed of one or more state requirements that are connected with logical operators AND or OR. States can only alter within the defined state space presented in Tables S3, S4. All reactions in the pathways that were implemented are executed asynchronously. Therefore, an induced state change has immediate effects on the next steps in the model. The algorithm stops if no operation causes a state change in any component anymore, thus the logical steady state is reached. From this information it can be concluded which genes are active or not. In summary, the presence or absence of nutrients leads to a cascade of events and finally expression or repression of target genes.

The model can optionally simulate knockouts or deletions of components. It is equivalent to setting the component's “presence” state in the model to 0. Consequently, such a perturbed component cannot participate in any operation in the model. The eliminated components are listed by their names and given as input to the model. All pathways are connected by crosstalk that can be manipulated in the model. The crosstalk reactions, listed in Table 2 and Table S8, can be switched on (1) and off (0) as a complementary input. By activating crosstalk, additional operations between proteins belonging to different pathways are appended.

The output is organized in tables sorted by pathways. In addition, separate tables are generated for the metabolites and for miscellaneous proteins that are shared over multiple pathways. Each component is part of exactly one table in which its steady state vector is given. Besides ordinary text files, a schematic picture of the cell for each pathway is created (Figures 3A–D, 4A). Moreover, an extra file with all involved genes and their final status as the output of the model is saved.

Furthermore, the model is designed in such a way that it can sequentially switch between input metabolites, i.e., from no glucose to glucose or vice versa. Under each nutrient condition the steady state is found and used as an initial condition for the next iteration. Outputs are generated after each step. The MATLAB code of the model and the simulations is provided at https://github.com/cvijoviclab/LogicModel.

### 2.2. Yeast Strains and Culture

The *S. cerevisiae* yeast strains were grown overnight to mid-log phase at 30°C in Yeast Nitrogen Base (YNB) synthetic complete medium containing 1.7 g/l yeast nitrogen base, 5 g/l ammonium sulfate, 670 mg/l complete supplement mix supplied with the appropriate amount of carbon source. All used strains in this work are summarized in Table S1.

### 2.3. Fluorescence Microscopy

The overnight culture grown on YNB supplemented with 4% glucose was diluted to an OD of 0.5 in either YNB media supplemented with 4% glucose or 3% ethanol depending on which environmental conditions was imaged. Fluorescent images were obtained by capturing 5 μl media between a microscopic slide and a cover glass. This was inserted in an inverted Leica DMI4000 microscope with a Leica CTR 4000 fluorescent light source and Leica DMI4000 Bright field light source operating on the LAS AF operating system (AF6000 E). Images were acquired using a HCX PL APO CS 100.0X1.40 oil objective with the LECA DFC360 FX camera. Exposure times used were 20 ms for the bright field state, 320 ms for the red fluorescent (mCherry) state, and 350 ms for the green fluorescent state (GFP).

### 2.4. Western Blot

The *S. cerevisiae* yeast strain was grown overnight to mid-log phase at 30°C in YNB supplemented with 6% glucose. The cultures were diluted 1:2 with fresh YNB media supplemented by either 4% glucose or 0.05% and incubated for 2 h at 30°C. Five milliliters was used for sampling. NaOH was added to a final concentration of 0.1 M and left for incubation at room temperature for 5 min. The samples were spun down and the pellet resuspended in 400 μl of 2M NaOH with 7% beta mercaptoethanol and incubated for 2 min. at room temperature. Four hundred microliters of 50% TCA buffer was added and the samples were spun down. The pellet was washed with 500μl Tris-HCl, resuspended in 50 μl sample buffer [62.5 mM Tris-HCL(pH = 6.8), 3% SDS, 10% glycerol, 5% beta mercaptoethanol] and boiled for 5 min at 100°C. Protein concentration was determined using DC™ Protein Assay, BioRad. Thirty microliters of 6 mg/ml protein was loaded on a 4–20% Mini-PROTEAN^®^ TGX Stain-Free™ Protein Gel, BioRad. The gel was imaged for full protein using Gel Doc EZ System, BioRad, and blotting was done using the Trans-Blot^©^ Turbo™ Transfer System, BioRad. The membrane was washed 3 x 5 min with 20 ml TBS buffer before blocking and after incubation with the antibodies. Blocking was done for 1 h using Western Blocker™ Solution for HRP detection systems, Sigma-Aldrich. The membrane was incubated for 1 h 15 min with Phospho-AMPKa (Thr172) (40H9) Rabbit mAb, Cell Signaling, diluted 1:1,000 and 1 h with TidyBlot, BioRad diluted 1:500. The membrane was imaged using ChemiDoc™ Imaging Systems, BioRad and SuperSignal™ West Pico PLUS Chemiluminescent Substrate, Thermo Scientific™.

## 3. Results

### 3.1. Vector Based Logic Modeling Allows for Modeling Protein States

Constructing the topologies of signaling networks is a challenging task, mainly because one protein can be in many different states, for example phosphorylation status and localization (Rother et al., 2013). In typical Boolean networks, nodes can only take the discrete values “0” and “1”, meaning a node is either inactive or active, and if active the signal is passed on to the next node. This approach does not allow for discrimination between multiple states of a node without introducing new nodes that would represent each single state. The complexity of the system would in this way be vastly increased. Therefore, an approach is required that allows the nodes of the model to be in several states. A multi-valued logical model is able to take into account several states (Abou-Jaoudé et al., 2016). However, this approach become impractical when there is a large amount of multiple states in which several states results in the same outcome. To overcome this obstacle we apply a vector format to a rules based model (Hlavacek et al., 2006; Boutillier et al., 2018). In a rule based model a reaction, defined as a state change of a node, only occurs given that certain exceeding rules or conditions are fulfilled. These conditions are defined as the required states of nodes for a reaction to be generated. Granted that no other reaction in the system will change this state, the node is in the logical steady state (LSS). We further assign every node, from here referred to as component, a component specific vector. In our modeling approach we distinguish between three different components: a protein component, metabolite component, and a complex formation component. The last component type is used for complex formation, and can only be “1” (active) or “0” (inactive). For metabolite and protein components a different vector format is used (Tables S2, S3). The vector for a protein component has 6 positions which describe the name, presence, localization, phosphorylation status, GDP/GTP exchange status, and DNA binding status of the protein. In the metabolite vector there are 3 positions which describe name, presence, and localization of the metabolite. For example, hypothetical signaling pathway X consisting of protein components Prt1 and Prt2 with the system only having one condition (Figure 1A). When simulating the system component Prt2 is initially not phosphorylated, therefore, position four in the component vector is “0.” When the conditions are fulfilled, namely both Prt1 and Prt2 are present in the system and Prt1 is phosphorylated, only then does position four in the vector for Prt2 change to “1”, meaning that the protein becomes phosphorylated. We used this framework to reconstruct a model describing glucose signaling networks derived from literature. The reconstruction included the Snf3/Rgt2, the Snf1 pathway and the cAMP-PKA pathway (Figure 1B). We manually mined the literature to find the components needed to connect the input conditions (“glucose” or “no glucose”) to the output gene expression. For yeast, glucose is a preferred carbon source since it can enter directly into the glycolysis after import into the cell. Therefore, yeast will prefer to metabolize glucose over other carbon sources. This model encompasses 48 components of which 45 are protein components and 3 are metabolite components (Table S2). All of these are unique proteins and metabolites except for the hexose transporters. Transporters Hxt1 to Hxt17 are a group of hexose transporters of which each has different glucose uptake characteristics (Kruckeberg, 1996; Horak, 2013). To reduce the complexity, we have grouped them together as one protein component named HXTs. The Rgt1 transcription factor becomes hyper-phosphorylated when the cell is exposed to glucose and is phosphorylated in a minor extent when glucose is not available (Flick et al., 2003). Therefore, we have chosen to assign the status of hyper-phosphorylated Rgt1 as “1” and “0” for the minor phosphorylated status in the component vector on the position for phosphorylation status. All the components in the model are divided into five different tables: metabolites, Snf1pw, R2S3Pathway, PKApw, and Miscl. The last table, Miscl, is for the metabolites and components of the Snf1 pathway, Rgt2/Snf3 pathway, cAMP-PKA pathway, and protein components belonging to neither or being shared over more than one of the previously named pathways. These tables are comprised of the component vectors. Further the model includes one complex component to signal the formation of an active PKA complex. Overall, the components take part in 61 rules or conditions (Table S5). This model reconstruction gives an overview of the connections between the involved components in glucose signaling reactions.

### 3.2. Gap Filling Processes Reveal a Lack of Protein Phosphatase Components and the Importance of Crosstalk From PKA Pathway

**Figure 2:**
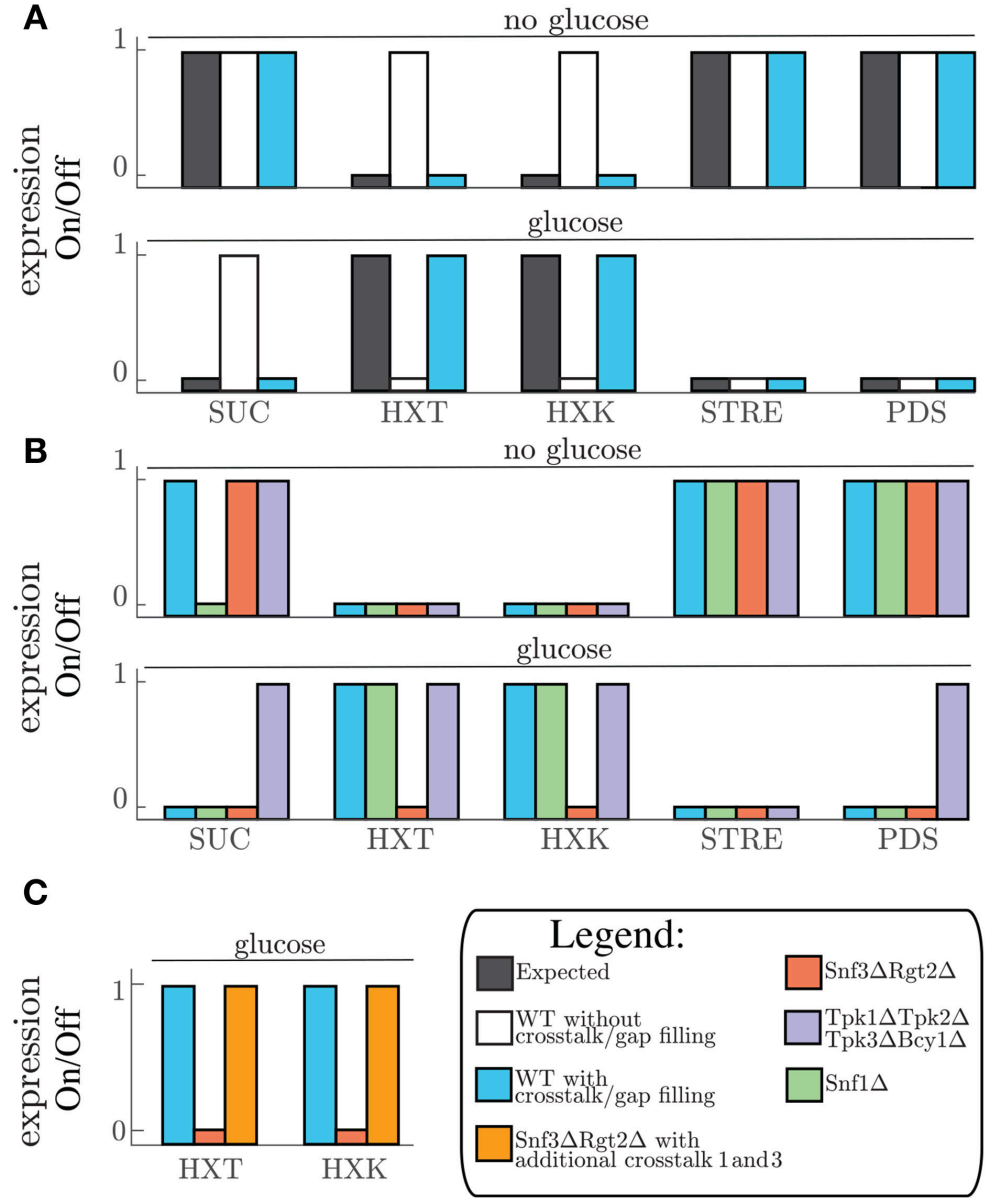
**(A)** Expected gene expression pattern (black, left) compared to the predicted gene expression state from the model without (white, middle) and with addition of crosstalk reactions 7 and 9 (Table 2), and after the gap filling process (Blue, right) for “no glucose” conditions (upper part) and “glucose” conditions (lower part) given for all the grouped genes. **(B)** Predicted gene expression state for WT-model (crosstalk reactions 7 and 9, and gap filling process) (blue,left) and the perturbations *snf1*Δ (green, middle left−side), *snf3*Δ*rgt2*Δ (red, middle−right side), and *tpk1*Δ*tpk2*Δ*tpk3*Δ*bcy1*Δ (purple, right) given for all the grouped genes. **(C)** Predicted gene expression state for WT-model (blue, left) compared to predicted gene expression states of *snf3*Δ*rgt2*Δ without (red, middle) and with crosstalk reaction 1 and 3 (orange, right) for the gene group HXT and HXK. SUC is the name for the gene SUC2. HXT is the group name for genes HXT1, HXT2, HXT3, and HXT4. HXK is the name for the gene HXK2. STRE is the group name for LSC1, PDC6, and PDC5. PDS is the group name for RHR2, HSP12, DDR2, and CCT1.

From this model we set out to make a system that can switch between “glucose” and “no glucose” as input conditions and make it reproduce the correct RNA expression profile as an output. To validate this we let the model reach the LSS for a certain condition after initialization and thereafter switch to the other condition. The original model from the literature reconstruction (Figure 1B) was able to correctly simulate the LSS for the first input conditions but unable to switch to the second expected LSS (Figure 2A). We therefore used the simulation to analyze what steps in the network are missing to successfully simulate the expected outcome. Additional unknown components needed to be added in the model to compensate for missing reactions that eventually lead to correct RNA expression profiles in all cases. This gap filling process was performed in an iterative model extension process suggested in an earlier study on carbon signaling pathways (Lubitz et al., 2015). To successfully reproduce the input/output of the network we added six additional conditions (Table 1). This resulted in the addition of four unknown protein components to the model. These unknown components were added to the table of miscellaneous protein components (Miscl). Interestingly, the first four gaps required the addition of a protein phosphatase component. The finding that four out of six unknown parts that needed to be added to the model contained protein phosphatases is intriguing. This suggests a general lack of knowledge about dephosphorylation processes of proteins in the glucose signaling network. The other two parts required the addition of a known crosstalk reaction from the PKA pathway to the Rgt2/Snf3 and Snf1 pathways (Table 2).

**Table 1:** Gap filling: Added parts after gap filling procedure in order to make the model switch between LSS for “glucose” and “no glucose” conditions.

**#**	**Involved components**	**Gap description**	**Added component**
1	Std1, Rgt1	Dephosphorylation of Std1 and Rgt1	Xxx1
2	Yak1, Rim15	Dephosphorylation of Yak1 and Rim5	Xxx2
3	Reg1, Glc7	Dephosphorylation of PP1 complex Reg1-Glc7	Xxx3
4	Msn2, Msn4	Dephosphorylation of Msn2 and Msn4	Xxx4
5	Glc7, Reg1	Phosphorylation of Glc7-Reg1	Crosstalk 7 (Table 2)
6	Rgt1	Phosphorylation of Rgt1	Crosstalk 9 (Table 2)

**Table 2:** Crosstalk: different types of crosstalk added to the model.

**#**	**Involved components**	**Description**	**Source**
1	Snf1, Mth1, Std1	Active Snf1 prevents inactivation of Mth1 and Std1	Gadura et al., 2006; Pasula et al., 2007
2	Snf1, Std1	Std1 stimulates the Snf1 kinase activity	Hubbard et al., 1994; Tomás-Cobos and Sanz, 2002; Kuchin et al., 2003
3	Reg1, Glc7, Yck1, Yck2	Reg1-Glc7 acts as an upstream activator of Yck1 and Yck2	Gadura et al., 2006
4	PKA complex, Sak1	PKA complex phosphorylates Sak1	Barrett et al., 2012
5	Snf1, PKA complex	PKA complex negatively regulates the Snf1 pathway (Sak1 independent)	Barrett et al., 2012
6	Snf1, Msn2	Snf1 can phosphorylate Msn2	De Wever et al., 2005
7	PKA complex,	glucose activation of the PKA complex pathway	Castermans et al., 2012
	Glc7, Reg1	is required for activation of PP1 (Glc7-Reg1)	
8	Snf1, Cyr1	Snf1 deactivates Cyr1 by phosphorylation	Nicastro et al., 2015
9	PKA complex, Rgt1	Bcy1 phosphorylates Rgt1 under high “glucose” conditions	Kim et al., 2006; Jouandot et al., 2011; Roy et al., 2013

### 3.3. Vector Format Boolean Network Simulation Can Predict and Visualize the State of Network Components

**Figure 3:**
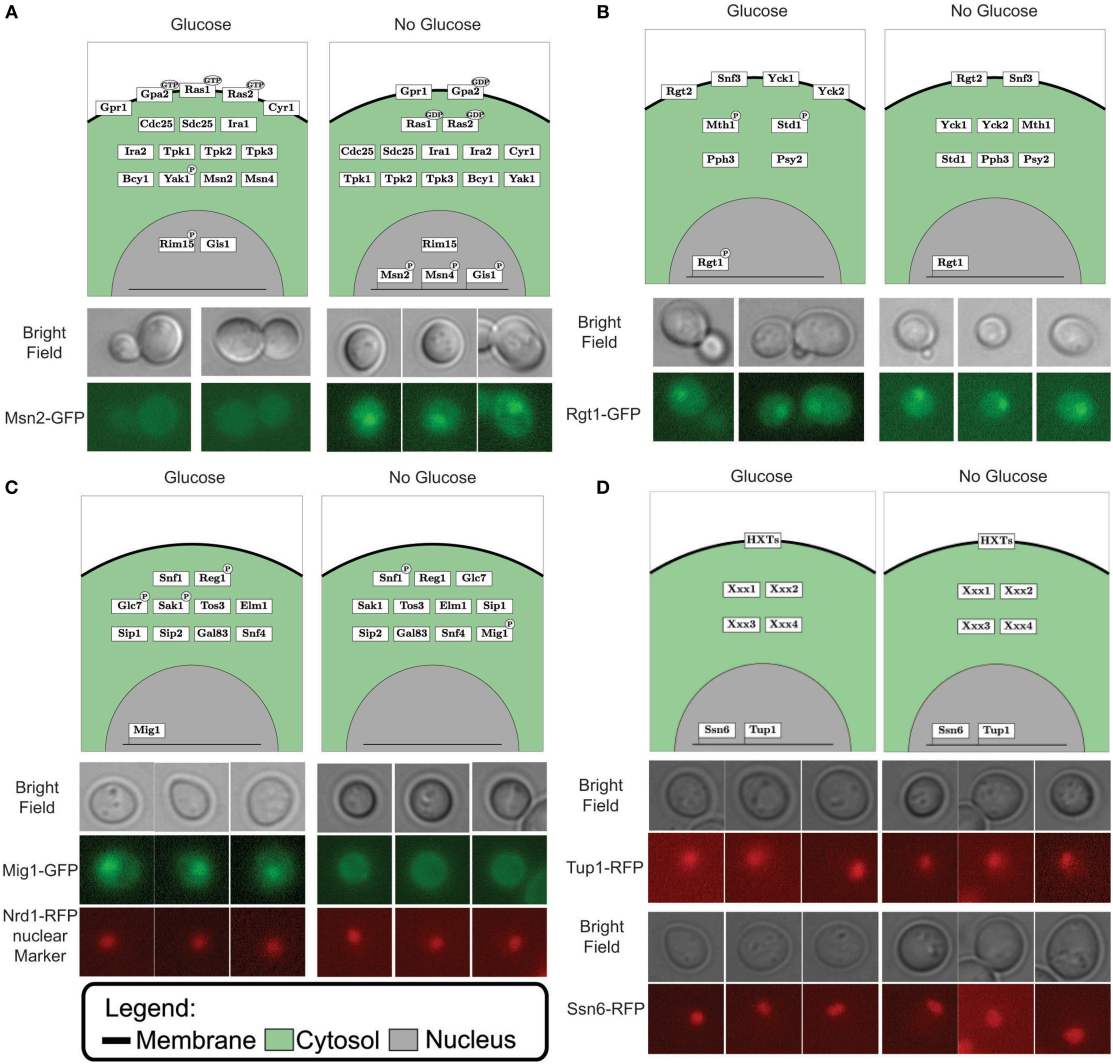
Simulated localization vs. microscopy data for Msn2 in the PKA pathway **(A)**, Rgt1 in the Rgt2/Snf3 pathway **(B)**, Mig1 in the Snf1 pathway **(C)** and both Ssn6 and Tup1 as general transcription repressors **(D)**. The upper part of the each panel displays the graphical representation of the simulated vector tables for each pathway. Protein location is depicting by the box in either the membrane (black line), cytosol (green area) or nucleus (gray area). Post-translational modification such as GDP/GTP binding and phosphorylation are displayed by a black ellipse or circle on the protein box. The DNA bound status is give by the protein box being connected to the line (which displays DNA). The lower part of each panel displays the microscopy data. The images above display the bright field, the lower images displays the fluorescent marked transcription factor for **(A,B)**. The middle image display the fluorescent marked Mig1 transcription factor and the lower image display Nrd1 bound with a red fluorescent protein used a marker for the nucleus **(C)**. Panel **(D)** displays the bright field images in the upper panel and the fluorescently marked general transcription repressors Ssn6 and Tup1 in the lower part.

After the gap filling process the model could simulate the switching between input conditions and predict the matching output status (Figure 2A). When predicting the outcome for one condition we initialize the model to the opposite condition first, since signaling networks are in place to sense changing conditions. Through model simulations we can test the effect of “glucose” and “no glucose” conditions on the model components. By plotting the component tables of these simulations in a graphical overview we create a coherent and legible way to view the pathway components and their different states (Figure 3). This neat overview simplifies comparison of the simulated LSS for the components with physical experiments. To show this feature we selected the transcription factors Msn2, Rgt1, and Mig1 to represent each pathway involved in glucose signaling and the general transcriptional repressors Tup1 and Ssn6. A version of these proteins, tagged with a fluorescent protein, was observed under the microscope in 4% glucose and in 3% ethanol as carbon source, representing “glucose” condition and “no glucose” condition respectively. Msn2, a transcription factor targeted by the PKA complex, localized to the nucleus with ethanol as carbon source and remained in the cytosol when exposed to glucose according to the model predictions (Figure 3A and Figure S2). When observing Msn2 labeled with a fluorescent green protein (GFP) in “glucose” conditions we detect a uniform distribution throughout the cell of the fluorescent signal from the GFP molecule. When the cells are grown in “no glucose” conditions the signal from the GFP molecule is no longer evenly distributed with the majority of signal focused in one part of the cell. This result indicates that Msn2 protein is localized in the nucleus. For Rgt1 the model prediction anticipates Rgt1 to be present in the nucleus for both “glucose” and “no glucose” conditions (Figure 3B and Figure S2). Because it either activates the HXT1 promoter in response to glucose availability (Mosley et al., 2003) or binds to the promoters of the hexose transporters to recruit transcription repressors when glucose is depleted (Kim et al., 2003; Broach, 2012). Observation of the yeast strain with GFP labeled Rgt1 showed that under both environmental conditions Rgt1 remained in the nucleus. As it has been shown in the literature and in our model predictions transcription factor Mig1 targeted by the Snf1 pathway. Mig1 is nuclear when the cell is exposed to glucose and remains in the cytosol when growing on ethanol (De Vit et al., 1997) (Figure 3C and Figure S2). A yeast strain with both Mig1 tagged with GFP and Nrd1, a protein that always resides in the nucleus, bound to a red fluorescent protein (RFP) was used to determine the localization of Mig1. We observed that under “glucose” conditions Mig1 co-localizes to the Nrd1-RFP signal, but under “no glucose” conditions it remains uniformly distributed throughout the cell. The transcription repressor complex Ssn6-Tup1 is either recruited by Mig1 under “glucose” conditions or by Rgt1 when the cells are not exposed to glucose (Treitel and Carlson, 1995; Roy et al., 2013) (Figure 3D and Figure S2). Indeed, it was observed that under 4% glucose and 3% ethanol both Ssn6 and Tup1 are localized in the nucleus. In addition to component localization, the model also considers post-transcriptional modifications such as phosphorylation. The phosphorylation state can be used to validate the model. Dephosphorylation of the protein Snf1 occurs when the cells are exposed to glucose and Snf1 becomes phosphorylated when grown on ethanol as sole carbon source. Typically, phosphorylation status of proteins is measured by Western blot. When looking at the phosphorylation status of Snf1 via Western blot we observed that the Snf1 phosphorylation status from the model predictions and experimental results are similar (Figure 3C and Figure S1). In general, this shows that the model prediction can be validated not only with the RNA expression but also through observation of localization and post-transscriptional modification.

### 3.4. Crosstalk Reactions From cAMP-PKA to Rgt2/Snf3 can Restore Perturbed Network Signaling

The gap filling process showed that crosstalk reactions were required in order for the model to switch from one condition to another. We therefore collected known crosstalk reactions from the literature and selected 9 crosstalk reactions to test in our model (Table 2). Next, we looked for crosstalk combinations that contribute to the robustness of the yeast cell carbon source sensing system. The carbon source sensing system was perturbed for each pathway by removing (a) key protein component(s) from the model simulation (analogous to protein deletion). From her on the wild-type model with the gap filling parts (Table 1) and crosstalk reaction promoting PKA-dependent phosphorylation of Glc7 and Rgt1 will be referred as the wild-type (WT) model. We always included these additions in the WT simulations since they were crucial to have the correct expected gene expression profile as simulation outcome (Figure 2A). When referred to the WT model we mean the model in which no protein components are removed from the simulation. Removing components leads to an activation of different set of reactions, which in turn alters the LSS. Consequently, the gene expression levels are changed compared to the original (i.e., WT) state (Figure 2B). For the Snf1 pathway we removed the Snf1 protein component and this perturbation is referred as *snf1*Δ. For the *snf1*Δ model simulation of the predicted gene expression state only changed for the SUC2 genes in the “no glucose” conditions compared to the WT model. Perturbation of the Snf3/Rgt2 pathway was performed by removing Snf3 and Rgt2 from the model, this model is referred to as *rgt2*Δ*snf3*Δ. This perturbation showed a different gene expression state for both expression of the HXT and HXK gene groups than the WT-model. Finally, for the disruption of the cAMP-PKA signaling all components of the PKA-complex were removed from the system (Tpk1, Tpk2, Tpk3, and Bcy1). This perturbed model was designated *tpk1*Δ*tpk2*Δ*tpk3*Δ*bcy1*Δ and displayed a different predicted gene expression pattern for the PDS genes compared to the WT (Figure 2B). Although the *tpk1*Δ*tpk2*Δ*tpk3*Δ*bcy1*Δ showed continuously active PDS gene group it did not for the STRE gene group. This is because of a gap filling part that was added that caused Msn2 and Msn4 dephosphorylation in “glucose” conditions (Table 1). This dephosphorylation part caused inactivation of Msn2 and Msn4 even when the inactivation of Yak1 and Rim15 was disrupted in the *tpk1*Δ*tpk2*Δ*tpk3*Δ*bcy1*Δ model. To find out which crosstalk reaction can overcome the consequences of signaling disruption the effect of crosstalk on the altered gene expression patterns was analyzed. This was done by simulating all possible combinations of crosstalk 1-6 and 8 from Table 2 in the “on” or “off” state. This resulted in 128 crosstalk combination vectors, which were used to activate crosstalk in the *snf1*Δ, the *rgt2*Δ*snf3*Δ, and the *tpk1*Δ*tpk2*Δ*tpk3*Δ*bcy1*Δ model. Simulations were only done for the environmental conditions that showed a different gene expression pattern, namely for *rgt2*Δ*snf3*Δ in “glucose”, *tpk1*Δ*tpk2*Δ*tpk3*Δ*bcy1*Δ in “glucose” and *snf1*Δ in “no glucose” conditions. Each crosstalk reaction is active in half of the simulated crosstalk combinations. Every time a crosstalk reaction was active it was scored whether the predicted gene expression pattern behaved as the WT model or the perturbed system with all crosstalk reactions inactive (Figure S3). For *tpk1*Δ*tpk2*Δ*tpk3*Δ*bcy1*Δ in “glucose” and *snf1*Δ in “no glucose” and “glucose” conditions no combination of crosstalk reactions was able to overcome the effects of the perturbation (Figures S3C–E). Crosstalk 1 and 3 were shown to overcome the disruption effect of *rgt2*Δ*snf3*Δ in “glucose” conditions with every crosstalk combination they were active in. Crosstalk 1 and 3 are connections between the Snf1 and Rgt2/Snf3 pathway. If we simulated the *rgt2*Δ*snf3*Δ model with the connections between the Snf1 and Rgt2/Snf3 pathway included we were able to restore the WT gene expression pattern again (Figure 2C and Figures S3A,B). Considering a perturbed model, the crosstalk reactions that could restore the gene expression to the pattern predicted by the WT model may contribute to the signaling robustness of the yeast cell *in vivo*.

### 3.5. Addition of the TOR Pathway to the Model Shows Inter-connectivity Between Nitrogen and Glucose Signaling

**Figure 4:**
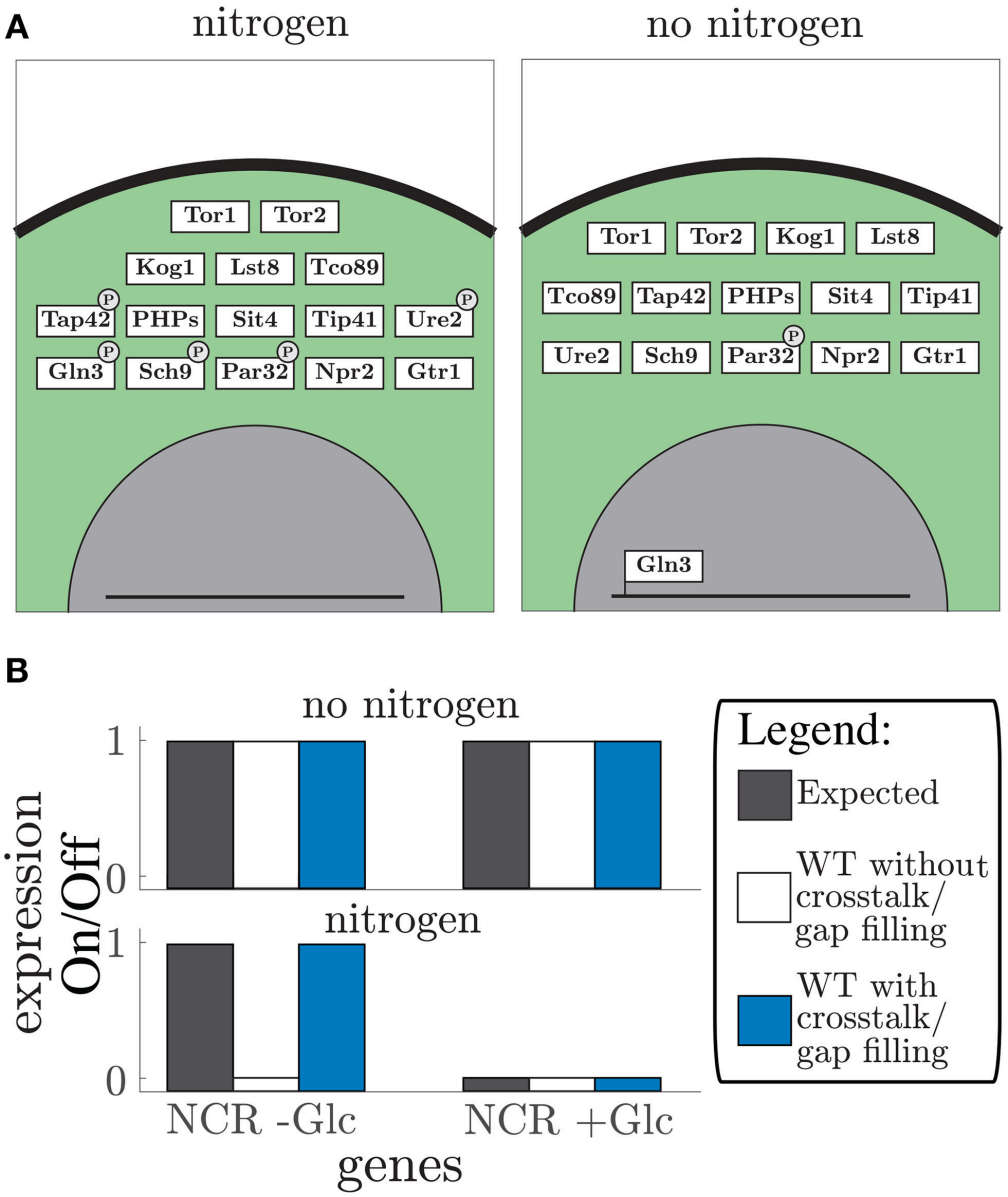
TOR pathway: **(A)** the graphical representation of the simulated vector tables for the TOR pathway. Protein location is given by depicting the box in either the membrane (black line), cytosol (green area), or nucleus (gray area). Post-translational modifications such as phosphorylation are displayed by a black ellipse or circle on the protein box. The DNA bound status is give by the protein box being connected to the line (which displays DNA). **(B)** Expected gene expression pattern (black, left) compared to the predicted gene expression state from the model without (white, middle) and with addition of crosstalk reactions, and after the gap filling process (blue, right) for “no nitrogen” conditions (upper part) and “nitrogen” conditions (lower part) given for the NCR genes in addition with and without glucose (Glc).

The vector format rule-based modeling allows the model to be altered by addition of single components or even new pathways. Here, we added regulation by the nitrogen sensing TOR pathway (Figure 4). The TOR pathway regulation is interesting to consider since glucose sensing pathways Snf1 and PKA-cAMP and the nitrogen sensing pathways TOR have shown to be highly intertwined (Broach, 2012; Sanz et al., 2016). Therefore, we added the nitrogen sensing pathway to our model focusing on the Sch9 and PP2A downstream targets. The TOR pathway includes 15 proteins and one gap filler which controls the NCR genes (Figure 4A). The TOR complex 1 (TORC1) was handled as the second complex component in the model. This expanded the model to 67 components of which 57 proteins, 4 metabolites, and 6 unknown components (Tables S6, S9), adding another 10 conditions to the Boolean model (Table S7). Furthermore, it led to four additional crosstalk reactions (Table S8), which connected glucose and nitrogen signaling. These connections converge on two components: Rim15 in the PKA-cAMP pathway and Gln3 in the TOR pathway (Rødkær and Færgeman, 2014). The model shows the importance of Snf1 in glucose starvation, specifically, through NCR gene expression in addition to nitrogen starvation through mediation of Gln3 nuclear localization. Thereby expressing NCR genes, during glucose limitation, even in nitrogen rich conditions. This crosstalk reaction allows the cells to use amino acids as an alternative nitrogen and carbon source (Bertram et al., 2002). Note that even though TOR and Snf1 dependent phosphorylation of Gln3 have different phosphorylation sites (Bertram et al., 2002), they are treated equivalently in the model. In both single cases and in the hyper-phosphorylated state it corresponds to a phosphorylation status “1” in the state vector. These phosphorylation sites are considered equivalent because they both cause Gln5/mediator interaction. After adding crosstalk the model was capable of simulating the expected gene expression of the NCR genes Bertram et al. (2002) (Figure 4B). The gap filling process led to two unknown components (Table S9) that are responsible for dephosphorylation of Kog1 and Par32. These additional parts are only affecting the outcome when crosstalk is present. Remarkably, similar to the glucose signaling, information about protein phosphatases is missing. Along with the increased size of the model, nitrogen availability was included as an additional input, allowing twice as many possible combinations of nutrient inputs. By adding the TOR pathway to the model we showed that the model is easily extended by single components and whole pathways due to the simple structure and modularity. Furthermore, the importance of crosstalk in signaling pathways shows the inter-connectivity of glucose and nitrogen signaling.

## 4. Discussion

To increase the information content of Boolean models from simple binary states, we assigned a vector to each component describing following features: localization, phosphorylation status, GDP/GTP exchange status, and DNA binding status (See section 2.1). Using this model, we found during the gap filling process that most lacking components are phosphatases, which indicates a lack of knowledge on phosphatases involved in nutrient sensing processes. The gap filling process also identified crosstalk from the PKA and Snf1 pathway to other pathways as a vital aspect to make the model switch between nutrient conditions. Model simulation of perturbed systems revealed that the crosstalk from the Snf1 pathway to the Rgt2/Snf3 pathway contributes to the robustness of this signaling network. The literature on nutrient sensing is quite extensive and this is a great resource to find mechanistic details on how the nutrient sensing network works. We set out to create a minimal system that can describe the RNA expression profile based on the input conditions. Most of the components and condition included in the model were shown in previous reports. However, for a few reactions different activation conditions were found, which are not mutually exclusive. Msn2 and Msn4 have been reported to be phosphorylated by Rim15, Yak1, and the PKA complex (Gorner et al., 2002; Lee et al., 2008, 2013). All these phosphorylation reactions have occurred in the active form of Msn2 and Msn4, although it is unclear which phosphorylation site(s) is/are deterministic for the function of Msn2 and Msn4. Since such reactions are closely related and appear almost simultaneously it is challenging distinguishing which reaction determines the occurrence of others, both computationally and experimentally. Such ambiguous mechanisms might result in multiple required conditions for a reaction to occur. All these conditions might not be representative *in vivo*, but do result in the same outcome as to be *in vivo* system. This is a limitation of modeling, since the model is only a representation of the knowledge we have of the system.

Since the knowledge gap in the literature did not allow us to create a model that could switch between nutrient conditions the gray areas needed to be filled in with a gap filling process. This network validation revealed that a common shortcoming on the knowledge of nutrient signaling pathways is how phosphate groups are removed from proteins, since the majority of the gaps in the model required addition of protein phosphatase reactions (Table 1 and Table S9). This led us to identify protein phosphatases as major unknown components of the glucose signaling pathways. The addition of a component does not necessarily mean a protein function is missing, also degradation of a phosphorylated component has been identified as a efficient phosphatase system (Rowland et al., 2015). Most studies on signaling pathways focus on phosphorylation of proteins, but for a precise regulation dephosphorylation most also be tightly regulated. However, research has been biased toward phosphorylation event and therefore dephosphorylation of proteins has received much less attention (Castermans et al., 2012). High-throughput studies have identified around 40 different proteins as protein phosphatase in *S. cerevisiae* (Fiedler et al., 2009). This overabundance and the overlapping function of these protein phosphatases has made the identification of the exact function of these phosphatases a challenging task. To illustrate, three different protein phosphatases have shown to be responsible for Snf1 dephosphorylation, namely the protein phosphatase complex 1 Reg1-Glc7, Sir4, and Ptc1 (Ruiz et al., 2011, 2013; Zhang et al., 2011; Castermans et al., 2012). It remains unclear how the two latter are regulated by glucose and what their direct function is in nutrient signaling. Also, only recently has the Glc7-Reg1 protein phosphatase complex been identified as the Mig1 glucose-dependent phosphatase, however there is also a glucose independent dephosphorylation mechanism which is unknown (Shashkova et al., 2017). The lack of knowledge on protein phosphatase function is not restricted to nutrient signaling, and is absent in other pathways in yeast (Sacristan-Reviriego et al., 2015).

During the gap filling process we also found that known crosstalk reactions needed to be added to fill gaps (Table 1). Since these mainly included the PKA pathway it is suggested that this pathway has established crosstalk toward other pathways. These connections might be vital for the correct functioning of the carbon sensing network. This explains the observation that most glucose-responsive genes are regulated by a PKA-dependent pathway (Wang et al., 2004). Further, the inviability of the *tpk1*Δ*tpk2*Δ*tpk3*Δ triple mutant indicates the important role of the PKA complex in the cell (Pan and Heitman, 1999). This shows the importance of the PKA pathway as regulator of carbon availability and suggests the PKA pathway as a possible intervention point for drugs targeting nutrient sensing in cancer cells. This was confirmed with recent publications suggesting that intervention in the PKA signaling pathway might prove to be a effective strategy to eliminate cancer cells (Klutzny et al., 2018; Le et al., 2018; Wu et al., 2018).

The crosstalk analysis shown here suggests that the Snf1 pathway interaction with the Rgt2/Snf3 pathway contribute to the robustness of nutrient signaling, since crosstalk was able to overcome the perturbation of the Rgt2 and Snf3 components (Figure 2C). This shows the overlap between the Snf1 and the Rgt2/Snf3 pathway. Earlier study on downstream targets of these pathways, namely Mig1 and Mig2, have shown a considerable overlap of targeted promoters (Westholm et al., 2008). Also the connection from the Snf1 pathway to the TOR pathway maintains correct balance in metabolism and shows how interaction between signaling pathways maintain signaling robustness in the cell. This study, together with others, has shown that pathways are not linear and do not exist parallel next to each other. There is a significant crosstalk between pathways, which is essential for the functioning of nutrient signaling (Zaman et al., 2008). Classically a sensing pathway is viewed as a singular element. However, it seems that sensing pathways reside within a large regulatory network, which overlaps between the different pathways.

Further, addition of other signaling pathways to our model is straightforward, which we demonstrated with the inclusion of TOR pathway. This opens the path of adding sensing and signaling mechanisms for other essential nutrients such as macro-nutrients phosphate and sulfate or micro-nutrients like metal ions (Conrad et al., 2014; Bird, 2015; Qi et al., 2016; Samyn and Persson, 2016). Potentially this could contribute to the understanding of how the cell senses macro-nutrients, which provide the cell carbon, nitrogen, phosphorus and sulfur, or micro-nutrients, such as metal ions and vitamins. The realization of this complete model would increase the perception of how nutrient sensing systems achieve sensitive cellular gene expression reprogramming.

The Boolean modeling system created in this work is discrete, deterministic, and semi-quantitative. This is an oversimplification of real sensing networks, but this problem could be overcome using a probabilistic Boolean modeling approach. This approach would be able to add molecular and genetic noise to the model (Liang and Han, 2012; Zhu et al., 2014), which would allow the input and output of the model to be continuous instead of discrete. This added complexity would result in a model that can provide more mechanistic detail. However, this would require a more complicated computational setup, which might prove to be a trade-off toward the modularity.

Overall, in this work we have developed, simulated and validated a Boolean logic model describing the nutrient sensing network in yeast. The development and validation process revealed the importance of crosstalk from one pathway to other nutrient sensing pathways and showed that the unknown components in the glucose signaling pathway are mostly phosphatases. By studying the interactions within the nutrient sensing network this work contributes to the holistic understanding of nutrient sensing and shows the impact of crosstalk on network robustness and functioning.

## Author Contributions

NW and MC conceived the presented idea. NW and BS designed the model and the implementation. BS performed the model simulations. NW and LÖ carried out the experiments. MC supervised the execution of the work. All authors discussed the results and contributed to the final manuscript.

### Conflict of Interest Statement

The authors declare that the research was conducted in the absence of any commercial or financial relationships that could be construed as a potential conflict of interest.
