# Supplementary material for: Robustness of Nutrient Signaling Is Maintained by Interconnectivity Between Signal Transduction Pathways
Source: Front Physiol. 2019 Jan 21;9:1964. doi: 10.3389/fphys.2018.01964 (PMC6348271; doi:10.3389/fphys.2018.01964)
Supplement: Supplementary file 1 [file Data_Sheet_1.pdf]

Supplementary:
Robustness of nutrient signalling is maintained by inter- connectivity between signal transduction pathways

Niek Welkenhuysen [1,2*], Barbara Schnitzer [1,2], Linnea Österberg [1,2,3] and Marija Cvijovic [1,2*]

[1] *Department of Mathematical Sciences, University of Gothenburg, Gothenburg, Sweden*
[2] *Department of Mathematical Sciences, Chalmers University of Technology, Gothenburg, Sweden*
[3] *Department of Biology and Biological Engineering, Chalmers University of Technology, Gothenburg, Sweden*

Correspondence*:
Marija Cvijovic
marija.cvijovic@chalmers.se

Niek Welkenhuysen
niek@chalmers.se

Strain name	background	genotype	sources
Rgt1-GFP	BY4741	MATa RGT1-GFP HIS3-MX6	[5]
YSH2350	BY47XX	MATa MSN2-GFP-HIS3 NRD1-mCherry-hphNT1 MET LYS	S. Hohmann collection
MCY14	W303-1A	NRD1-mCherry- Hph MIG1-GFP-KanMX	M. Cvijovic collection
YSH2796	W303-1A	MATa TUP1-mCherry-HPH	S. Hohmann collection
YSH2797	W303-1A	MATa SSN6-mCherry-HPH	S. Hohmann collection

Table S1. List of all strains and plasmids used in this study. BY47XX is the haploid strain from the cross between BY4741 and BY4742

Protein/Metabolite	Identifier in model	component type
Glc	Metabolites{1, : }	Metabolite
ATP	Metabolites{2, : }	Metabolite
cAMP	Metabolites{3, : }	Metabolite
Snf1	Snf1pw{1, : }	Protein
Reg1	Snf1pw{2, : }	Protein
Glc7	Snf1pw{3, : }	Protein
Sak1	Snf1pw{4, : }	Protein
Tos3	Snf1pw{5, : }	Protein
Elm1	Snf1pw{6, : }	Protein
Sip1	Snf1pw{7, : }	Protein
Sip2	Snf1pw{8, : }	Protein
Gal83	Snf1pw{9, : }	Protein
Snf4	Snf1pw{10, : }	Protein
Mig1	Snf1pw{11, : }	Protein
Rgt2	R2S3pw{1, : }	Protein
Snf3	R2S3pw{2, : }	Protein
Yck1	R2S3pw{3, : }	Protein
Yck2	R2S3pw{4, : }	Protein
Mth1	R2S3pw{5, : }	Protein
Std1	R2S3pw{6, : }	Protein
Rgt1	R2S3pw{7, : }	Protein
Pph3	R2S3pw{8, : }	Protein
Psy2	R2S3pw{9, : }	Protein
Gpr1	PKApw{1, : }	Protein
Gpa2	PKApw{2, : }	Protein
Ras1	PKApw{3, : }	Protein
Ras2	PKApw{4, : }	Protein
Cdc25	PKApw{5, : }	Protein
Sdc25	PKApw{6, : }	Protein
Ira1	PKApw{7, : }	Protein
Ira2	PKApw{8, : }	Protein
Cyr1	PKApw{9, : }	Protein
Tpk1	PKApw{10, : }	Protein
Tpk2	PKApw{11, : }	Protein
Tpk3	PKApw{12, : }	Protein
Bcy1	PKApw{13, : }	Protein
Rim15	PKApw{14, : }	Protein
Yak1	PKApw{15, : }	Protein
Msn2	PKApw{16, : }	Protein
Msn4	PKApw{17, : }	Protein
Gis1	PKApw{18, : }	Protein

Protein/Metabolite	Identifier in model	component type
HXTs	Miscl{1, : }	Multiple proteins
Ssn6	Miscl{2, : }	Protein
Tup1	Miscl{3, : }	Protein
Xxx1	Miscl{4, : }	Protein
Xxx2	Miscl{5, : }	Protein
Xxx3	Miscl{6, : }	Protein
Xxx4	Miscl{7, : }	Protein

Table S2. Glucose signalling model components. Table with all components, their model identifier and type of component.

Protein state	position in vector	possible states
protein name	XXXpw{ : , 1 }	
presence	XXXpw{ : , 2 }	(0) protein not present in system<br>(1) protein present in system
localization in the cell	XXXpw{ : , 3 }	(0) in or near cell membrane<br>(1) in cytosol<br>(2) in nucleus
phoshporylation status	XXXpw{ : , 4 }	(0) phosphorylation site not phosphorylated<br>(1) phosphorylation site phosphorylated
guanylation status	XXXpw{ : , 5 }	(0) no guanylation sites<br>(1) guanylation site bound with GDP<br>(2) guanylation site bound with GTP
DNA binding status	XXXpw{ : , 6 }	(0) protein is not DNA bound<br>(1) protein is DNA bound

Table S3. Protein component vector states: all position in the vector for protein components. Their model identifier in MATLAB and the possible states the position in the vector can take. XXXpw refers to any given pathway included in the model.

Metabolite state	position in vector	possible states
metabolite name	XXXpw{ : , 1 }	
presence	XXXpw{ : , 2 }	(0) metabolite not present in system<br>(1) metabolite present in system
localization in the cell	XXXpw{ : , 3 }	(1) in cytosol<br>(2) in nucleus

Table S4. Metabolite component vector states: All position in the vector for metabolite components. Their model identifier in MATLAB and the possible states the position in the vector can take. XXXpw refers to any given pathway included in the model.

Rule description	Glucose	PubMed ID
Hexotransporters import glucose to the cytosol, ATP is available in the cytosol	1	9299703
Reg1 dephosphorylation by unkonwn Xxx3	0	
Constituative Snf1 phosphorylation by either Sak1, Elm1 or Tos3		15831494, 17991748
Dephosphorylation of Snf1 by Glc7 and phosphorylated Reg1		15831494
SNF1 kinase complex beta and gamma subunits (Sip1, Sip2 or Gal83, and Snf4) are needed for active (phosphorylated) Snf1 and phosphorylate Mig1		2557546, 10990457
Mig1 dephosphorylation by Glc7 and phosphorylated Reg1		28854669
Unphosphorylated Mig1 is nuclear and DNA bound		9832517
Phosphorylated Mig1 is localted in the cytosol		
Ssn6 and Tup1 are recruited by DNA bound Mig1		7724528
Repression of the SUC, MAL and GAL genes by DNA bound Mig1, Ssn6 and Tup1		9832517, 9973625
Rgt2 and Snf3 are glucose sensors and act through Yck1 or Yck2 recruitment to the membrane	1	8901598, 14755054
Yck1 or Yck2 close to the membrane are responsible for activation (phosphorylation) of Mth1 and Std1		14755054
Inactive (dephosphorylated) Mth1 or Std1 leads to hyperphosphorylation of Rgt1		12925759
Mth1 dephosphorylation by Psy2 and Pph3	0	24277933
Std1 and Rgt1 dephosphorylation by unknown Xxx1	0	
Dephosporylation of Rgt1 returns its DNA binding capacity		12861007
DNA bound and dephosphorylated Rgt1 recruits Ssn6 and Tup1		
Repression of HXK2 and HXTs by dephosphorylated Rgt1, Ssn6 and Tup1 at the DNA		7862149, 12527758, 15705057
Hyperphosphorylated Rgt1 acts as a activator of HXTs		12527758, 26205245
Gpa2,the beta subunit of Gpr1, is associated with GTP when exposed to glucose	1	17983752
Gpa2-GTP interacts with Cyr1 at the membrane		17983752
Ras1 and Ras2 are associated with GTP by Cdc25 or Sdc25 if glucose is available intracellular	1	9628870, 8206969
Ras1-GTP and Ras2-GTP are anchored to the cytoplasm side of the cell membrane		8430318
Cyr1 localizes to the plasma membrane if there is Ras1-GTP or Ras2-GTP		1875942
Ras1 and Ras2 are GDP-loaded by Ira1 and Ira2 if glucose is depleted	0	15339905, 1668647

Rule description	Glucose	PubMed ID
Glucose addition (ATP) and Cyr1 at the plasma membrane cause a spike in cAMP	1	9628870
PKA complex is active if there is Bcy1 and Tpk1, Tpk2 or Tpk3 with cAMP		
Active PKA complex inactivates Rim15 by phosphorylation		16759348, 15661010
Active PKA inactivates Yak1 by phosphorylation		21255108
Activation (dephosphorylation) of Rim15 and Yak1 by unknown Xxx2	0	
Dephoshporylated Rim15 or Yak1 phosphorylates Msn2 and Msn4		18793336, 24140345
Active PKA complex represses nuclear localization of Msn2 and Msn4		9472026, 16281053
If PKA complex is inactive Msn2 and Msn4 are in the nucleus		9472026, 16281053
Inactive Rim15 deactivates Gis1 (connected to Igo1/2, Cdc55/Pph21/Tpd3 complex) by dephosphorylation and loss of DNA bound		23273919
Unknown Xxx4 dephoshorylates Msn2 and Msn4	1	
Dephosphorylated Rim15 stimulates Gis1 by phosphorylation		15300954, 10835355
Phosphorylated and nuclear Msn2 or Msn4 bind to the DNA and activate STRE genes		8650168, 8641288
Phosphorylated and nuclear Gis1 bind to the DNA and activate PDS genes		22363679

Table S5. Rules: All rules/conditions implemented in the model. The glucose column indicates if the glucose conditions (high = 1, low = 0) is directly involved in the reaction. Crosstalk is separately described in Table 2.

Protein/Metabolite	Identifier in model	component type
NH3	Metabolites{4, : }	Metabolite
Tor1	TORpw{1, : }	Protein
Tor2	TORpw{2, : }	Protein
Kog1	TORpw{3, : }	Protein
Lst8	TORpw{4, : }	Protein
Tco89	TORpw{5, : }	Protein
Tap42	TORpw{6, : }	Protein
PHPs	TORpw{7, : }	Multiple proteins (Php21/22/3)
Sit4	TORpw{8, : }	Protein
Tip41	TORpw{9, : }	Protein
Ure2	TORpw{10, : }	Protein
Gln3	TORpw{11, : }	Protein
Sch9	TORpw{12, : }	Protein
Par32	TORpw{13, : }	Protein
Npr2	TORpw{14, : }	Protein (also Npr3)
Gtr1	TORpw{15, : }	Protein (also Gpr2)
Xxx5	Miscl{8, : }	Protein
Xxx6	Miscl{9, : }	Protein

Table S6. TOR pathway components: Table with all added components belonging to the TOR pathway, their model identifier in MATLAB and type of component.

1 TOR PATHWAY

Rule description	Nitrogen	PubMed ID
Par32 phosphorylation by Gtr1 and Npr2	0	25085507
TOR complex formation (Tor1 or Tor2, dephosphorylated Kog1, Lst8, Tco89) TORC1	1	22964838
Sch9 phosphorylation by active TORC1		22964838
Tap42 phosphorylation by active TORC1		22964838, 22174183
Phosphorylated Tap42 recruits PHPs and Sit4 and is bound to the vacuolar membrane and can therefore not dephosphorylate Gln3 and Ure2		12820961, 25085507
Sch9 dephosphorylation through inactive TORC1		22964838
Tap42 dephosphorylation by inactive Sit4, PHPs and TOR complex		22964838, 22174183, 24738657
Activation of unphosphorylated Tap42 and Sit4 and PHPs by release in the cytosol by phosphorylated Par32 and inactive TOR complex		12820961, 20093466
Tip41 inhibits interaction of free cytosolic Tap42 with Sit4 and PHPs		11741537
Free cytosolic Sit4 and PHPs dephosphorylate Gln3 and Ure2		11741537, 10940301
Dephosphorylated Gln3 moves to the nucleus		22174183,22964838
Nuclear Gln3 expresses NCR gene		19104072, 20378536
Active TORC1 phosphorylates Gln3 and Ure2 in the cytosol		10940301

Table S7. TOR pathway rules: All added rules/conditions connected to the TOR pathway implemented in the model. The nitrogen column indicates if the nitrogen conditions (high = 1, low = 0) are directly involved in the reaction. Crosstalk is separately described in Table S8.

#	involved components	description	source
10	Snf1, Gln3<br>Sch9, Par32	Snf1 phosphorylates Gln3 and Kog1<br>and dephosphorylate Sch9 and Par32	[1, 8, 4, 3]
11	Xxx5	an unknown protein can dephosphorylate Par32<br>besides Snf1	[3]
12	Sch9, Tor, Rim 15, Msn2	active Sch9 or Tor represses Rim15, Tor represses<br>nuclear localization of Msn2 independently	[2, 7, 6]
13	Sch9, Gis1	active Sch9 phosphorylates Gis1 independent of Rim15	[2]

Table S8. TOR pathway related crosstalk: Different types off TOR related crosstalk added to the model.

#	involved components	gap description	Added component
6	Par32	Dephosphorylation of Par32	Xxx5
7	Kog1	Dephosphorylation of Kog1	Xxx6

Table S9. TOR pathway gap filling steps: Added TOR related gap filling parts in order to make the model switch between LSS states for ”nitrogen” and ”no nitrogen” conditions.

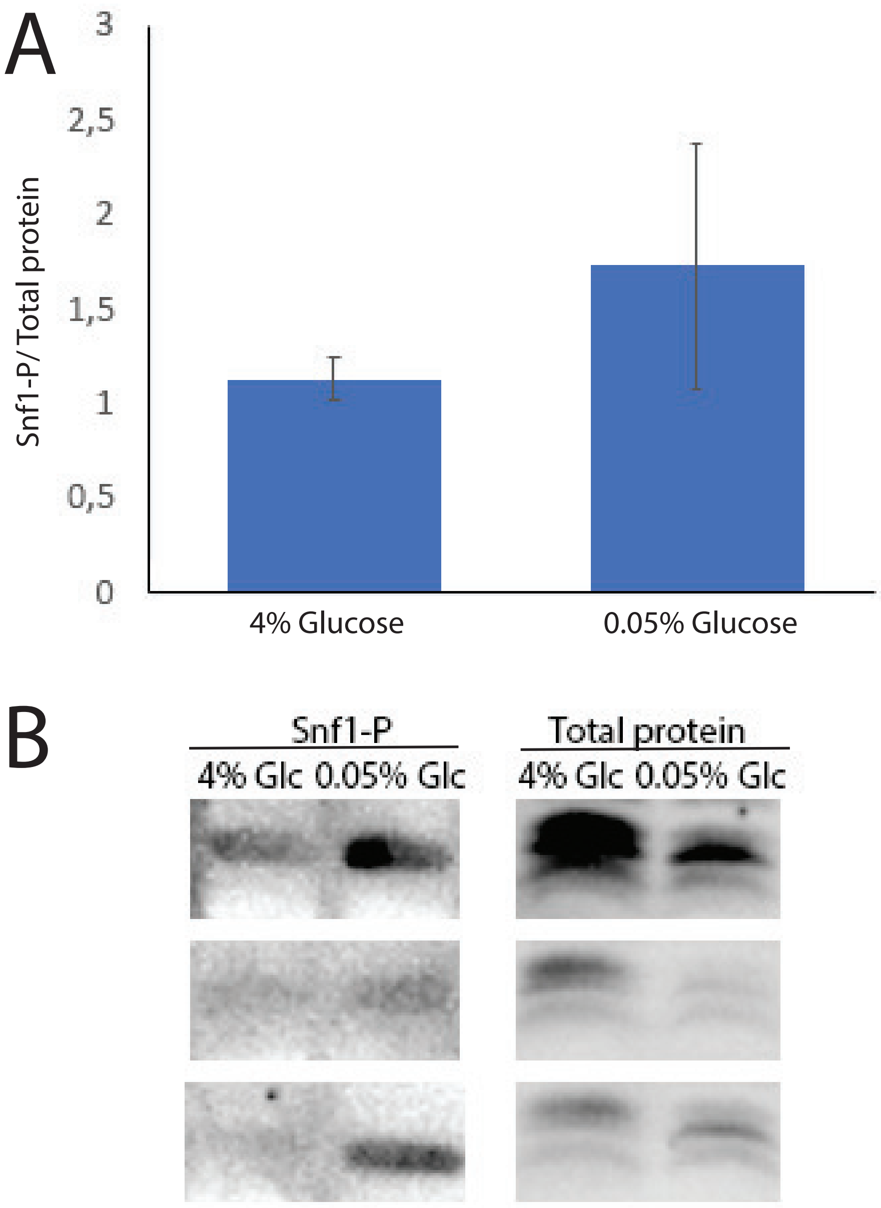

Figure 1. Snf1 phosphorylation in ”glucose” or ”no glucose conditions”. (**A**) Snf1 phosphorylation 2 hours after cells were incubated with either 4% glucose or 0.05% as ”glucose” and ”no glucose” conditions, respectively. Measurement of Snf1 phosphorylation is given by the intensity of the Anti-Snf1-P antibody divided by the total protein. Average is for three experiments, error bars are standard error. (**B**) Western blots of all three experiments. Left side is the Anti-Snf1 antibody staining, right side is total protein in Glucose conditions (4% Glc) and Low glucose conditions (0.05% Glc).

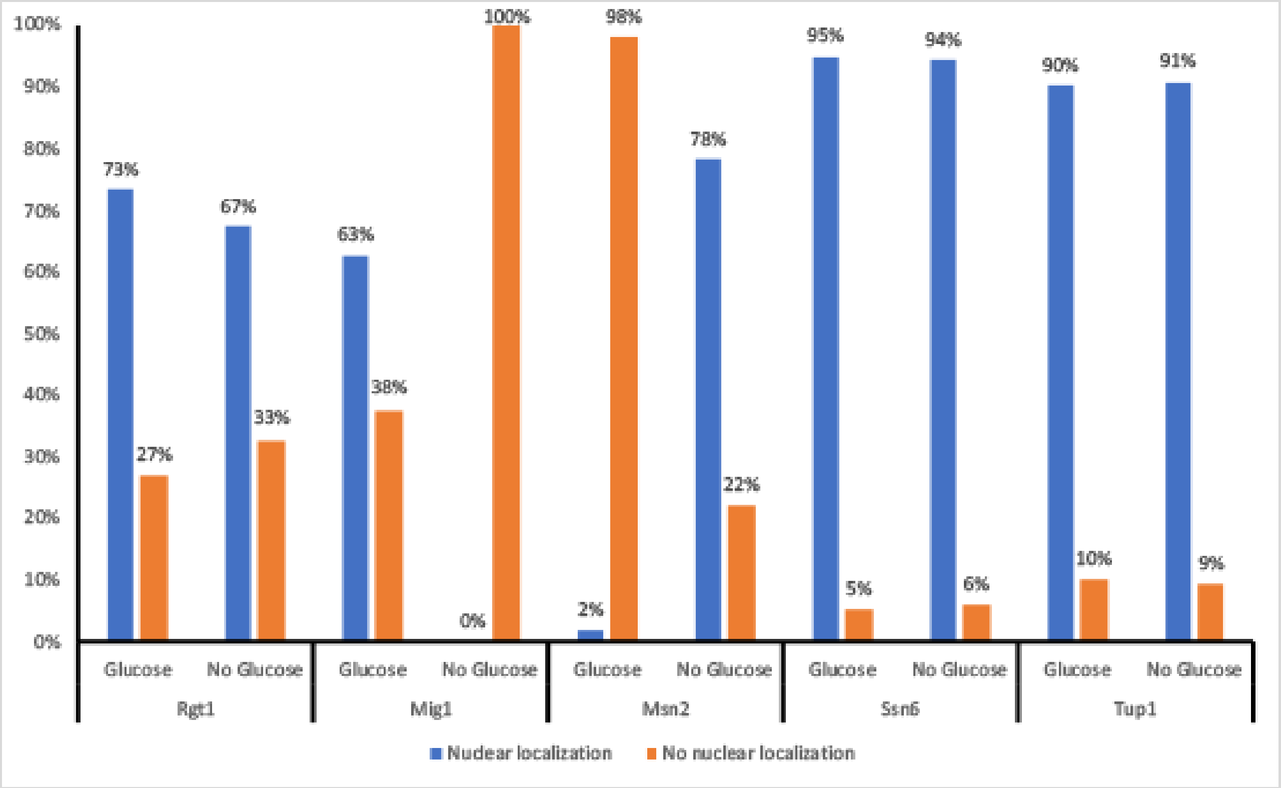

Figure 2. Distribution of nuclear localization determined from fluorescent data. Percentage of cells with nuclear localization (blue, left) and without nuclear localization (orange, right) of Rgt1, Mig1, Msn2, Ssn6 or Tup1. For each conditions for each strain between 32 - 62 cells were counted.

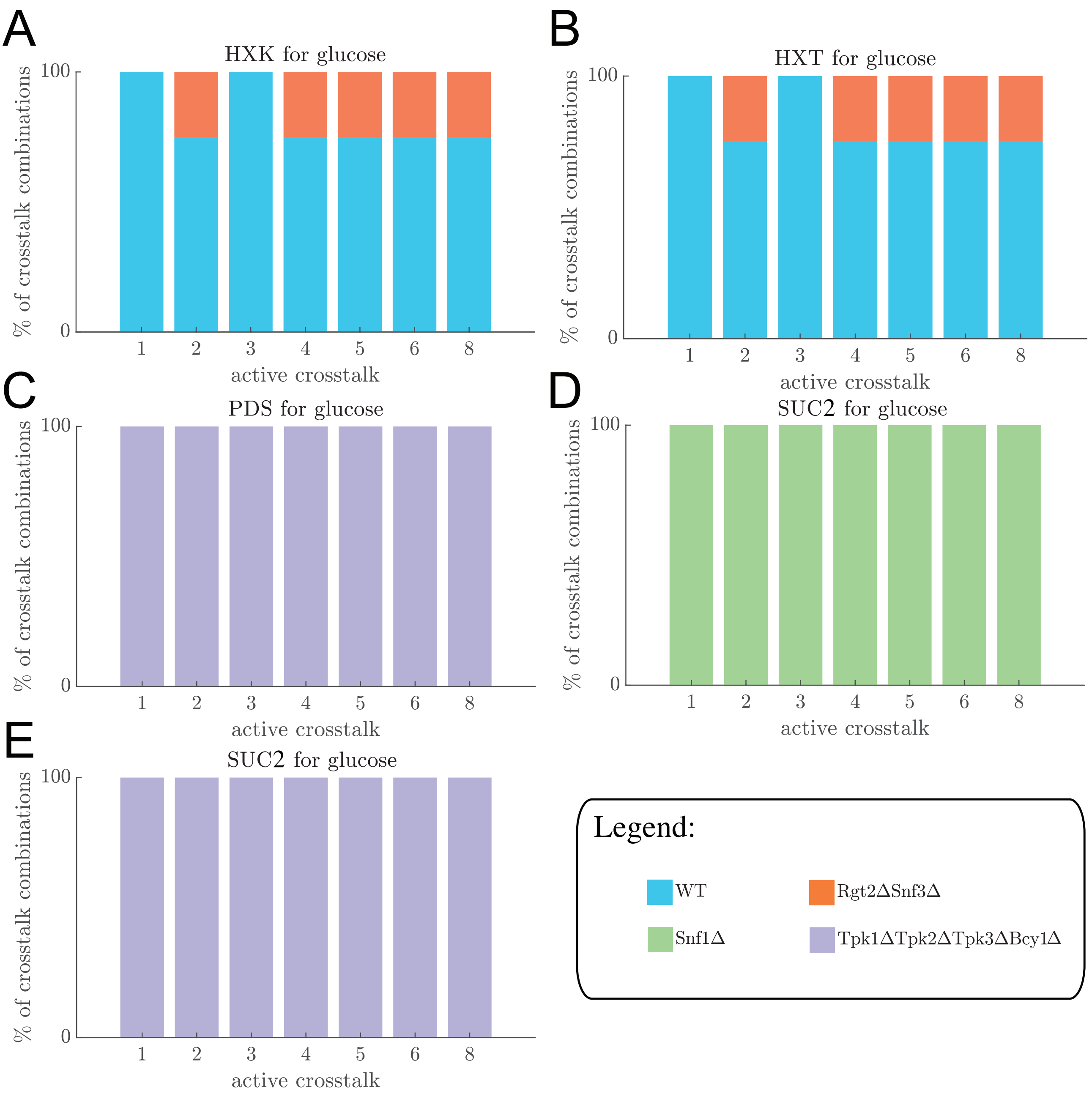

Figure 3. Crosstalk analysis of all possible combination of crosstalk 1-6 and 8 in cases of a model perturbation. Plot displays in which percentage of the cases a certain crosstalk (participating in the crosstalk combinations) mimicked the gene expression state for the WT-model or the perturbed model. (**A**) The HXK gene group for ”glucose” conditions, WT vs. *rgt2*Δ*snf3*Δ. (**B**) The HXT gene group for ”glucose” conditions, WT vs. *rgt2*Δ*snf3*Δ. (**C**) The PDS gene group for ”glucose” conditions, WT vs. *tpk1*Δ*tpk2*Δ*tpk3*Δ*bcy1*Δ. (**D**) The SUC2 gene group for ”no glucose” conditions , WT vs. *Snf1*Δ. (**E**) The SUC2 gene group for ”glucose” conditions, WT vs. *tpk1*Δ*tpk2*Δ*tpk3*Δ*bcy1*Δ. SUC2 is the group name for the gene SUC2. HXT is the group name for genes HXT1, HXT2, HXT3 and HXT4. HXK is the name for the gene HXK2.

REFERENCES

[1] Bertram, P. G., Choi, J. H., Carvalho, J., Chan, T.-F., Ai, W., and Zheng, X. F. S. (2002). Convergence of TOR-nitrogen and Snf1-glucose signaling pathways onto Gln3. *Molecular and cellular biology* 22, 1246–52. doi:10.1007/978-3-319-43589-3_7

[2] Fabrizio, P., Pozza, F., Pletcher, S. D., Gendron, C. M., and Longo, V. D. (2001). Regulation of longevity and stress resistance by Sch9 in yeast. *Science (New York, N.Y.)* 292, 288–290. doi:10.1126/science.1059497

[3] Hughes Hallett, J. E., Luo, X., and Capaldi, A. P. (2014). State transitions in the TORC1 signaling pathway and information processing in Saccharomyces cerevisiae. *Genetics* 198, 773–786. doi:10.1534/genetics.114.168369

[4] Hughes Hallett, J. E., Luo, X., and Capaldi, A. P. (2015). Snf1/AMPK promotes the formation of Kog1/Raptor-bodies to increase the activation threshold of TORC1 in budding yeast. *eLife* 4. doi:10.7554/eLife.09181

[5] Huh, W.-K., Falvo, J. V., Gerke, L. C., Carroll, A. S., Howson, R. W., Weissman, J. S., et al. (2003). Global analysis of protein localization in budding yeast. *Nature* 425, 686–691. doi:10.1038/nature02026

[6] Pedruzzi, I., Dubouloz, F., Cameroni, E., Wanke, V., Roosen, J., Winderickx, J., et al. (2003). TOR and PKA signaling pathways converge on the protein kinase Rim15 to control entry into G0. *Molecular cell* 12, 1607–1613

[7] Swinnen, E., Wanke, V., Roosen, J., Smets, B., Dubouloz, F., Pedruzzi, I., et al. (2006). Rim15 and the crossroads of nutrient signalling pathways in Saccharomyces cerevisiae. *Cell division* 1, 3. doi:10.1186/1747-1028-1-3

[8] Tate, J. J., Rai, R., and Cooper, T. G. (2005). Methionine sulfoximine treatment and carbon starvation elicit Snf1-independent phosphorylation of the transcription activator Gln3 in Saccharomyces cerevisiae. *The Journal of biological chemistry* 280, 27195–204. doi:10.1074/jbc.M504052200
